# Parental Perspectives of the Impact of COVID-19 Lockdown on Food-Related Behaviors: Systematic Review

**DOI:** 10.3390/foods11182851

**Published:** 2022-09-15

**Authors:** Elzbieta Titis

**Affiliations:** Warwick Institute for the Science of Cities, 4th Floor, Mathematical Sciences Building, University of Warwick, Coventry CV4 7AL, UK; e.titis@warwick.ac.uk

**Keywords:** food, feeding style, eating trends, food interactions, food intake, food preparation, food management, food insecurity, meal planning, parent, child, family food environment, COVID-19, systematic review

## Abstract

Home confinement during the COVID-19 pandemic has been accompanied by dramatic changes in household food dynamics that can significantly influence health. This systematic literature review presents parental perspectives of the impact of COVID-19 lockdown (up to 30 June 2022) on food preparation and meal routines, as well as other food-related behaviors, capturing both favorable and unfavorable changes in the household food environment. Themes and trends are identified and associations with other lifestyle factors are assessed. Overall, families enjoyed more time together around food, including planning meals, cooking, and eating together. Eating more diverse foods and balanced home-cooked meals (e.g., fresh fruit and vegetables) was combined with overeating and increased snacking (e.g., high-calorie snacks, desserts, and sweets), as parents became more permissive towards food; however, food insecurity increased among families with the lowest income. Adoption of meal planning skills and online shopping behavior emerged alongside behaviors aimed at self-sufficiency, such as bulk purchasing and stockpiling of non-perishable processed foods. These results are an important first step in recognizing how this pandemic may be affecting the family food environment, including low-income families. Future obesity prevention and treatment initiatives, but also ongoing efforts to address food management, parental feeding practices, and food insecurity, can account for these changes moving forward.

## 1. Introduction

The outbreak of coronavirus disease (COVID-19) in late December 2019 in China, which later developed into a long-enduring pandemic, resulted in unprecedented changes to civil and social activity on a global scale, causing not only a health crisis, but also a series of issues pertaining to social, economic, and food security aspects [1]. To contain the spread of the disease, governments’ responses around the world included strict lockdowns or curfews, reliance on quarantine, and adherence to social distancing. Large-scale social restrictions included schools switching to distance learning, work from home, keeping at least one meter from each other, places of entertainment being closed, cancelation of public events, and closing of international borders and airports, to name a few [2]. This changed routine activities around the globe, such as those relating to daily shopping or within the transportation sector; as a result, the deficit in the retail system in the first wave of COVID-19 took place alongside consumers making the rapid shift to online services. Many other social, psychological, and economic challenges followed, including all aspects of food-related decisions and behaviors [3].

As a result of quarantine and social isolation, access to fresh food has been limited, mainly due to difficulties in transportation, distribution, and delivery [4]. Moreover, quarantine and social distancing may encourage consumers to favor ultra-processed food that have longer shelf life [5,6], or they may stimulate unhealthy eating through inducing emotional disturbance, boredom, stress, and anxiety [7,8,9]. The impact of the pandemic and containment measures also led to a severe contraction in economic activity and resulting loss of disposable income, having a devastating impact on food poverty levels and inequality; as a result, “the proportion of people who could not afford even half the cost of a healthy diet increased from 43% pre-COVID-19 (2020) to 50%” during the lockdown [10]. On the other hand, the pandemic has the potential to encourage positive changes in eating behavior, such as spending more time with family around food or eating together more home-cooked meals as opposed to eating out; additionally, people may also engage in health-seeking behaviors, including healthy eating, to seek protection from COVID-19 [11]. Interestingly, one study has shown that changes due to the pandemic were in line with pre-pandemic goals, such as favoring more local production, choosing unpacked or recyclable/biodegradable packed foods, or paying attention to one’s weight; therefore, the pandemic may have been a catalyst for behavioral change [12].

The focus of this review is on individual- and community-level challenges as experienced in the family setting during the COVID-19 pandemic. The importance of the family food environment in establishing healthy eating habits during childhood/adolescence is well understood and includes considerations for both home food availability and parental modelling of dietary behaviors [13]. Recently, the pandemic directly impacted household activities, including work, eating food away from home, grocery shopping, and childcare [14], which in turn have influenced diet quality and decreased food waste [14]. Results for the former are mixed, as, for example, increased time spent in preparing food at home has been related to higher diet quality [14]; however, one systematic review of longitudinal studies found that the pandemic has led to increased alcohol consumption, snack frequency, and a preference for sweets and ultra-processed food rather than fruits, vegetables, and fresh food [15]. Because a large number of studies have been conducted to assess these changes (some of which providing mixed results), this review adds value by providing a clear summary for both researchers to inform future research and limit duplication, and practitioners who will be more aware of the impacts that their patients/clients may be experiencing.

Specifically, the aim is to synthesize the available evidence on parental perspectives of the impact of COVID-19 lockdown on the family food environment and food-related activities, capturing both favorable and unfavorable changes in food preparation and meal routines, as well as other relevant behaviors revolving around food, such as food shopping, meal planning, and eating habits. Other reviews in the food domain have mainly been concerned with eating behavior changes in the general population [15,16], including low- and middle-income countries [17], adherence to the Mediterranean diet [18], obesity risk factors [19], the relationship between dietary intakes and immunity [20], effects on diet and physical activity in older adults [21], and parental perceptions of the food environment and their influence on food decisions among low-income families [22]. According to author’s best knowledge, this is the first systematic review that aims to comprehensively study the evidence relating to general parental food perspectives during the pandemic, including low-income parents. Assessing the parental perspective is appropriate as parents have higher food involvement owing to the need to provide for the family, and the burden falls on them. Results can inform policy and interventions in relation to promoting long-term adoption of improved food purchasing/management and feeding practices in the family setting. 

## 2. Materials and Methods

All articles that examined parental/caregiver perspectives on the family food environment/food-related activities during the COVID-19 pandemic, which included meal preparation and other family interactions around food (e.g., conversations, gardening, cooking, and eating together), were considered eligible for this review. A literature search of the PubMed, Scorpus, and Web of Science databases was conducted up to 30 June 2022 using the following terms: (Eating OR feeding OR eating behavio* OR eating habits OR eating trends OR food OR food choices OR food consumption OR diet* OR dietary trends OR dietary patterns) AND (COVID-19 OR lockdown OR pandemic) AND (child* OR adolescen* OR parent* OR caregiver OR family) AND (cook* OR food preparation OR meal preparation OR food shopping OR meal planning)’. References of eligible studies and relevant reviews were also searched, using a snowballing technique.

Results were screened for eligibility based on title, abstract, and finally full text. The inclusion criteria of this literature search included the following:

1. Limit to papers published up to 30 June 2022 (including pre-prints);
2. Studies that investigated the association of COVID-19 lockdown and parental/caregiver perspectives of family interactions around food, including food preparation and meal routines;
3. The age range including children and adolescents, along with their parents/caregivers;
4. Changes in family interactions around food could be reported by children/adolescents or by parents/caregivers;
5. Only research articles in English.

There were no restrictions other than those stated in the inclusion criteria above. Consequently, there was no demographic restriction other than age, and all study designs were considered eligible. The review discusses all the research articles published during the lockdown phases as identified by the literature search up to the time specified, offering a global overview from several countries. Literature, systematic, or narrative studies reviewing previous research were excluded using automatic search limits in databases; additionally, studies that considered chosen aspect(s) of parental perspectives on the family food environment (e.g., dietary patterns) in isolation from meal preparation and family interactions around food were also excluded from the synthesis.

For final full-text studies included in the review, the following characteristics were extracted: first author, year, title, journal, objective, type of study, method, participant number, age, location, findings, and conclusion. These results are shown in Table S1 in the Supplementary Materials. Additionally, rapid qualitative analysis methods were used to identify themes around changes in parental perspectives on the family food environment/food-related activities, and data from each paper for the relevant themes were gathered and grouped together for analysis. Results were summarized via a narrative review; a quantitative synthesis was not attempted due to the heterogeneity of the samples and methodology between studies.

The study was performed according to the Preferred Reporting Items for Systematic Reviews and Meta-Analyses (PRISMA) guidelines [23].

## 3. Results

**Figure 1:**
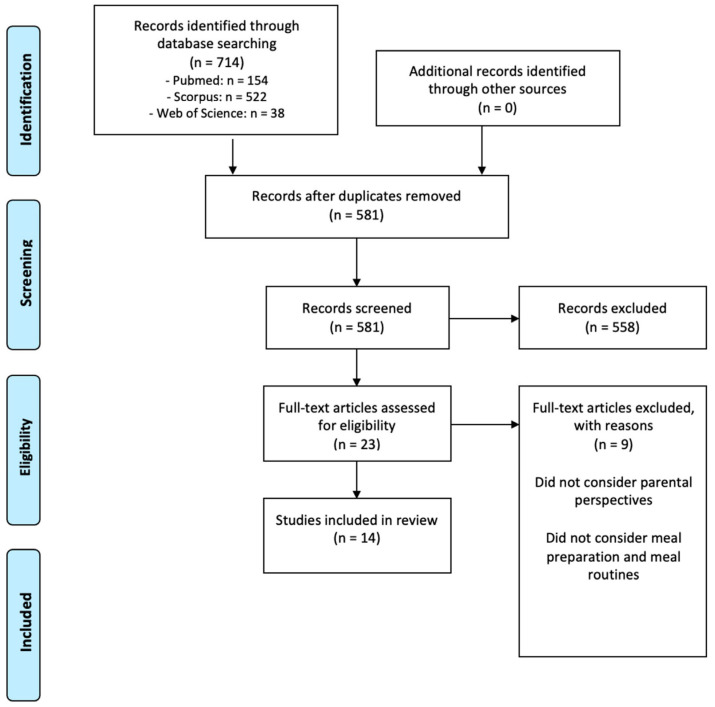
PRISMA 2009 flow diagram displaying the selection process of the 14 final papers.

Out of a total of 581 papers initially identified (after removing duplicates), 14 full papers were included in the synthesis (see Figure 1). Included studies were from different countries, thereof four European and four American; other locations included Canada, Mumbai in India, Gaza Strip in Palestine, and Australia, whereas two studies were focused on more than one country and included New Zeeland and China. Eight studies used quantitative surveys, four studies used qualitative interviews, and two studies used both quantitative and qualitative data (surveys with closed-ended questions analyzed via descriptive statistics and open-ended responses analyzed thematically). These results are summarized in Table S1 in the Supplementary Materials.

The results suggest that nutrition-related changes occurred during the lockdown in both unfavorable and favorable directions. Table 1 summarizes the main results regarding the research themes: food purchasing and planning, meal preparation and routines, and eating and feeding behaviors. Subsequently, thematic synthesis yielded the following three themes of changes: ‘changes in meal planning and shopping behaviors’, ‘changes in food preparation behaviors and meal routines’ and ‘changes in feeding and eating behaviors’. Each theme was analyzed from the parental perspective and reflects a stage of the decision-making process around food that directly affects children’s eating practices. In addition, considerations about weight/obesity and the uneven burden of COVID-19 on families are also addressed, as they were important areas of interest found in the studies (*n* = 6). The following discussion is separated into four parts to support both the research themes and additional considerations.

### 3.1. Changes in Meal Planning and Food Shopping Behaviors

General trends in meal planning and grocery shopping behaviors during the pandemic were adoption of meal planning skills [27,31,33], shopping less often [26,31], observing prices going up [31], shortage of food items [27,30], need to provide a nutritious diet for health [28,29,31], and shift towards home food deliveries [26,27,31]. Three studies did not report on this aspect of food-related perspectives [24,32,34].

**Figure 2:**
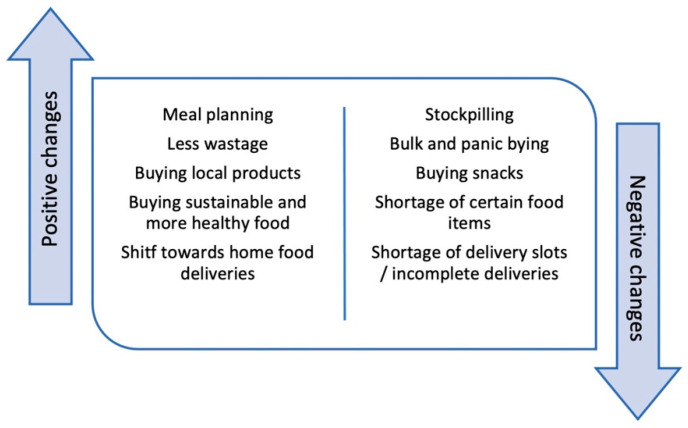
Positive and negative changes in food planning and shopping.

Negative changes included stockpiling shelf stable foods due to grocery shortages [30] and to minimise social exposure [37]; this included bulk [27] and panic buying [37], and purchasing more sugary drinks (SDs) and snacks [30], high-calorie snacks, desserts and sweets [36], and non-perishable processed foods [36]. Others were critical of bulk buying [31] or appreciated choices of more fresh, seasonal, and local foods [29], the latter of which was often related to an increased choice of fruits and vegetables [29]. Food planning improved [27] in terms of providing an all-inclusive balanced diet to keep family members strong and healthy [31]; positive food attitudes in the more prudent use of food with less wastage were also observed [35]. Parents more frequently bought foods their child liked [28], sought their children’s opinions about what they would like to eat for the meals [33], or got their children involved in menu planning [32,33]. Parents also bought more healthy and sustainable foods [28], the former which resulted from emerging nutrition concerns [29]. One study reported some concerns about frequency of grocery shopping as parents would like to shop less often but fresh produce may not last that long [25]. Moreover, food-related support, such as help offered by friends, food donations and school meal assistance, was critical for economically disadvantaged families, who also reported increased importance of food offers during lockdown, difficulty in finding delivery slots, and incomplete food deliveries [31]. Figure 2 shows the discussed changes to meal planning and shopping behaviors.

### 3.2. Changes in Food Preparation Behaviors and Meal Routines

**Figure 3:**
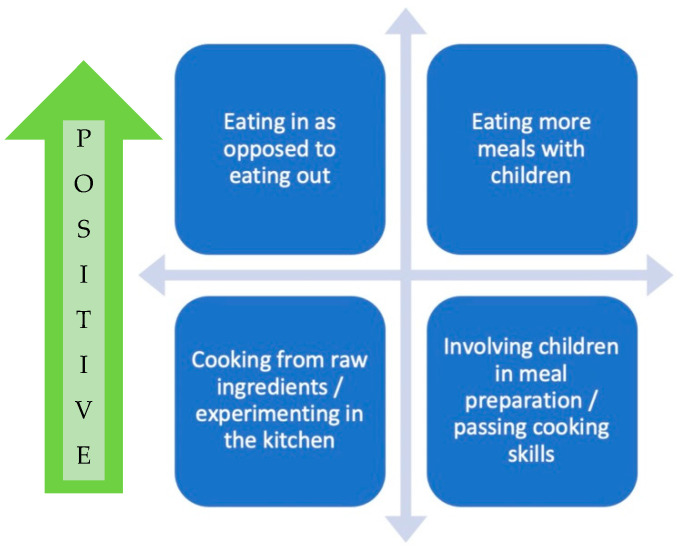
Positive changes in food preparation and routines.

The majority of parents reported that their meal routines had changed for the better since COVID-19; favorable changes to their meal routines included eating home-cooked meals as opposed to eating out, eating more meals with children, and involving children in meal preparation. These positive trends were reported in all the studies and no negative changes were observed. Moreover, parents made healthier choices as a result of not being “on the go” [30], including making more meals from scratch [25] and experimenting in the kitchen [27,29]. Cooking with the child was a pleasurable activity [29,32] and an occasion to educate about food, to pass on certain cooking skills and values around food, and to taste new flavors [29]; additionally, as part of the meal preparation routine, parents also involved their children in gardening together [32,33]. One study reported that cooking skills confidence was associated with a higher frequency of including children in cooking activities, and that a higher intake of vegetables by parents was predictive of more frequent inclusion of children in cooking activities [24]. Others found that being a female parent predicted a higher/lower frequency of the child consuming home-made meals/food at restaurants [26]. Figure 3 shows the discussed changes to food preparation behaviors and meal routines.

### 3.3. Changes in Feeding and Eating Behaviors

The impacts of COVID-19 on feeding behaviors in parents and subsequent eating behaviors in their children have been varied. Many positive changes in feeding and eating practices were observed during the lockdown, including increased consumption of hot and home-cooked lunches [32,33], and greater concern for health and immunity starting to impact food choices [27]; consequently, lunch quality also improved, including increased variety, more elaborate and complex meals, and healthier meals [32,33]. Moreover, eating at a calm pace had positive consequences for the meal atmosphere at home and on children’s eating behaviors [29], and being home together, made some parents more aware of their children’s SD consumption and/or help them to control it [30]. Children were also more interested in and likely to accept food they helped to prepare themselves [29]. On the other hand, changes in children’s daily routines during the COVID-19 pandemic had negative impacts on their eating behavior, such as overeating and eating frequency [25,27,28,35], increased intake of sugary drinks (SDs) [30] and snacks [29,30,34,37], and emotional eating [28,31]; one study also reported that children were skipping breakfast when attending school virtually [30]. Moreover, these negative changes in nutrition were accompanied by a more flexible and lenient parenting style, which further facilitated unhealthy eating in children [28,30,31,34]. One study did not report on this aspect of food-related parental perspectives [24].

**Figure 4:**
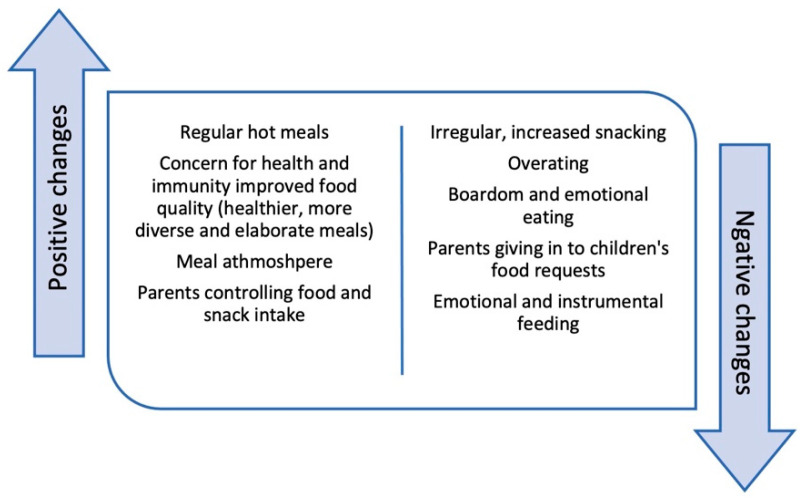
Positive and negative changes eating and feeding behaviors.

Several factors were thought to fuel an excess consumption of SDs and snacks in children during the pandemic, including unrestricted access, boredom, and a lack of mealtime schedule and structure [30]. Parents were more likely to eat with their younger children, providing more structure around meals and restricting snacks; however, younger children were also more often subjected to instrumental feeding and emotion-based snack feeding by their parents [34]. Others argue that the majority of children had regular mealtimes but irregular snack times [34], and that parental stress may be further responsible for non-nutritive use of food and snacks [34], resulting in emotional and instrumental feeding [28,34]. The pandemic also altered parents’ oversight of children’s SD and snack consumption, such that parents became more lenient [31] and permissive [28], allowing their children more autonomy in making their own decisions about food [28,30] and giving into children’s food requests [31]. In one study, removing prior restrictions on SDs was also justified by parents as a coping strategy to help children deal with change [30]. On the other hand, not all parents showed this amount of tolerance with food and were involved in controlling the food or snack intake of their children [30,33,34]. Figure 4 shows the discussed changes to eating and feeding behaviors.

### 3.4. Additional Considerations about Weight and Uneven Burden of COVID-19 on Families

Five studies included considerations regarding weight and/or obesity [29,34,35,36,37], and one study examined the impact of COVID-19 on the food decisions of poor families [31]. Overall, the total amount of food in the home increased [36,37], and people reporting weight gains outnumbered those reporting weight loss [35]. Others argue that more parents reported obesity in their children after lockdown [37], or that they had some concerns about their children’s weight [29,36]. Food security status defined the amount of change in food intake, including the total amount of food that was eaten, fresh and non-perishable foods, and desserts and snacks [36]; also, families that were food insecure had more concerns about child being overweight but still urged their children to eat more than their body required, compared with food-secure families [36]. One study also found that, for low-income families, the family’s food needs had increased and infrequent shopping trips and reliance on supermarket home deliveries compromised opportunities to continue eating fresh food products [31]; consequently, food-related support was critical during the COVID-19 lockdown/s, especially among single-parent families, who may also have had more challenges than parents living with partners in adopting healthy eating habits [31].

**Figure 5:**
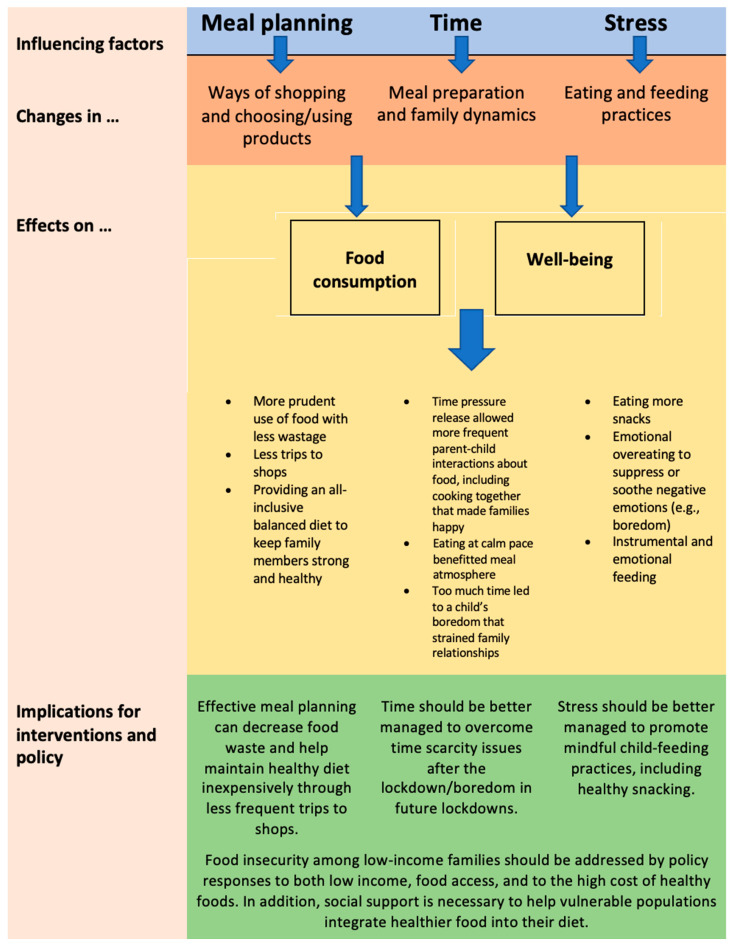
Changes in food-related behaviors, their determinants, and implications.

Figure 5 shows highlighted changes in food-related behaviors in relation to their determinants and implications for interventions and policy, which is discussed in more detail in the next section.

## 4. Discussion

This systematic review provides summaries of peer-reviewed published evidence on parental perspectives of the impact of COVID-19 lockdown on food preparation and meal routines; additionally, other aspects of food-related behaviors in the family setting are also addressed, including meal planning and grocery shopping, and feeding and eating practices. Overall, parents had many enjoyable interactions with their children about food during the lockdown, which seemed to have a positive impact on cooking (e.g., cooking home-made foods, increased intention to cook from raw ingredients, children becoming more involved in the meal preparation) and food planning (ensuring healthy eating, less trips to the shop, more prudent use of food and less wastage). Almost all of the included studies showed frequent consumption of well-balanced homemade meals, but also overeating and increased unhealthy snacking reinforced by more flexible and lenient parenting style. New trends in grocery shopping, weight concerns, and the uneven burden of COVID-19 on families were also reported. The following discussion addresses these issues in more detail using headings chosen for the purpose of best illustrating identified trends.

### 4.1. More Time Allows Family to Enjoy Food and Moments Together, but Also Leads to Boredom

Time was cited as a factor that gave families the opportunity to plan meals and moments together [29,35], to prepare diverse and well-balanced meals [29,30], and to eat at a calm pace [29], the latter having positive consequences for the meal atmosphere at home and on children’s eating behaviors [29]. On the other hand, increases in food responsiveness and emotional overeating were significantly correlated with an increase in child boredom at home [28,30]. 

Family interactions and engagement are crucial for the family to eat healthily, as eating practices are intricately tied to family life, and people tend to eat healthier when eating together with their family [38]. Specifically, family awareness has been found to help plan meals and facilitate social comparison [38]; this includes snacking awareness prompting caregivers to prepare snacks ahead of time for their children and purchase healthier foods for the home [38]. Before the lockdown, time was an important barrier for most parents, especially those working full time [39]. Indeed, time can be thought of as a health resource, as, for example, lack of time is the main reason people give for not taking exercise or eating healthy food [40]; time pressure is also negatively and consistently associated with mental health over time [41]. Moreover, the evidence suggests that time pressures contribute to socially patterned health inequalities among people caring for others [40]; for example, single mothers who are both time- and income-deprived may face compounding barriers to good diet and health [40]. To face time pressures, parents often resort to meal simplification or taking out, losing sight of what is nutritionally beneficial [42]; in order to balance healthy meals with time constrains, meal planning [43] and time management [44] have been recommended as suitable strategies. On the one hand, strategies to manage time scarcity are needed to further promote and facilitate family engagement around food after the lockdown, including home-based food preparation; on the other hand, social policies and planning and health interventions should continue involving the time dimension to minimize time–income–space trade-offs faced by individuals [40].

On the other hand, given the recent lockdown, a new phenomenon of time abundance appears to be as damaging to healthy eating as time pressures, as children who are bored at home resort to emotional eating (EE) [28]. Individuals with EE use eating to reduce the intensity of negative emotions [45]; this provides instant gratification [9], but is a poor coping strategy leading to more eating [46]. It is also possible that difficulties in emotion regulation may be one possible mechanism underlying EE [47]. Since eating in response to negative emotions involves consumption of palatable foods to lift the mood [9], EE may predict weight gain in adults [48]. Moreover, the clustering of health behaviors in children [49,50] raises the question of whether EE is also related to physical activity (PA), sedentary behavior (SB), and/or sleep duration [46]. Previous results suggest that boredom is an important construct that should be considered a separate dimension of emotional eating [51]. Moreover, short-term effects of the COVID-19 pandemic on PA and SB in children have been observed, which may become permanently entrenched if proper measures are not taken into account [52]. Evidence shows that positive family environments could help children cope with unexpected disturbances in their daily life under lockdown; however, the emerging weariness and boredom reported by some children in the second wave of the lockdown strained family relationships [53]. Moving forward, in addition to promoting PA and reducing SB in children, programmatic and policy strategies should focus on time management skills, including educating parents and children on how to manage free time to continue having positive family interactions and combat excessive boredom in children.

### 4.2. Health and Immunity Determined the Food Preparation and Intake

Some parents mentioned that, because of the lockdown, they became interested in the nutrition and motivated to provide a diversity of foods and balanced meals [29]. For some, fruit and vegetables became an important component of a healthy diet, and thus always featured in food shopping lists [25], as opposed to basing food choice around food preferences and taste. Others wanted to buy fresh produce, but worried about shelf life as they would prefer to shop less frequent [25]. This desire to eat more healthily during the pandemic was stimulated by either altered perceptions of health and immunity [27], or more choices of fresh, seasonal, and local foods on the market [29].

Experiences from previous outbreaks have shown that during the “life” course of an epidemic, people’s concerns about health and immunity grow stronger for self-protective motives [11]. Most of the nutrition and dietary recommendations to combat viral infections, including COVID-19, revolve around maintaining a balanced diet [8], as existing evidence highlights that nutrients play an essential role in immune cell triggering, interaction, differentiation, or functional expression [54,55,56], thus having a profound effect on people’s immune system and disease susceptibility. Research conducted during the COVID-19 outbreak alerted people to the importance of nutrition in protecting people’s health in times of pandemic [57], reporting the link between the levels of various nutrients and the severity of symptoms in COVID-19 [58,59,60], or relating diet-related ill-health (e.g., obesity) to a worse prognosis for the disease [61]. Studies that examined the relationship between COVID-19 fear during lockdown and family food habits have identified increasing needs to provide an all-inclusive balanced diet for growth and health [28], including an increased choice of fruits and vegetables [29,31]; however, despite the best intentions to eat more healthily in times of the pandemic, some parents described issues of grocery shortages, leading to the making of different food than was originally intended (e.g., whole green gram pulse instead of buying vegetables) [27], whereas others turned to stockpiling shelf stable foods [30], the latter being also driven by concerns about fresh food preservation [25]. On the other hand, for low-income families, it was the reliance on supermarket home deliveries that compromised home food availability in fresh food products [31].

In addition, a related line of research has examined pandemic-induced stress and food-related mental health [62,63,64,65]. Previous research has revealed a wide range of psychosocial impacts of infectious disease that may produce fear in the community or individuals in relation to getting sick or dying, or feeling helpless or stigmatised [66]. As a result, the pandemic may lead to fear-induced eating disorders [66], and recent evidence attributes increased fear and worries caused by the pandemic to eating pathologies in children [67] and caregivers [63]. Additional efforts are needed to maintain this level of public focus on diet-related health and immunity after the lockdown; in addition, education about preserving the shelf life of fresh produce could prepare the public for better managing food in future outbreaks.

### 4.3. Frequent Consumption of Homemade Meals, but Also Increased Unhealthy Snacking

Most parents observed an increase in the overall food intake of their children during the remote learning period [32], who also ate more home-cooked and hot meals [32,33]; additionally, as a result of cooking more meals at home, food quality also improved [33], as meals became more varied and healthier [28,33]. On the other hand, the increased amount of food in the household had negative impacts on food consumption patterns in children, such as overeating or increased eating frequency and snacking [27,35], which led some parents to express concerns about their children weight [29,37]. 

Evidence shows that cooking dinner frequently at home is associated with consumption of a healthier diet [68]; as a result, meal preparation at home is increasingly being promoted as an obesity reduction measure [69,70]. On the other hand, the consumption of food prepared away from home is associated with a lower quality diet and a higher body mass index (BMI) [71,72]. Still, healthy cooking depends on an individual’s ability to use healthy ingredients and techniques (e.g., grilling or steaming vs. deep frying or sautéing) [68]. As degradation of traditional cooking skills progresses [73], meals at home often include processed foods with 36% of dishes being purchased in their finished form or finished entirely to package directions [74]. The exceptional circumstances of the lockdown provided a positive opportunity for more cooking among the general population [75], including cooking from raw ingredients [31], which, overall, was associated with eating more fresh products, including fruits and vegetables [75], and thus better diet quality and health status. Others, however, reported a decline in their diet quality due to consumption of comfort food and snacking [75,76,77], or food supply issues [75]. Research conducted during the COVID-19 pandemic supports previous evidence linking mood states with eating behaviors [78,79]. For example, a French study showed that mood was associated with the increased intake of processed meat and sweet-tasting and alcoholic beverages during the pandemic [78], whereas in Italy, comfort eating and overall increase in food intake was observed to improve the sense of wellbeing [79].

These negative trends in eating behavior during the lockdown may be particularly problematic because the increased consumption of “comfort” foods was combined with the dramatic reduction in energy expenditure, leading to energy imbalance and thus to weight gain [80]. Indeed, evidence shows that a significant proportion of the population gained weight during the lockdown [81]. Changes in cooking frequency also varied among population subgroups, as individuals in financial difficulty tended to cook less [75]; in this sense, the lockdown increased social health inequalities. Previous research suggests that healthier dietary alternatives are available even in low resource areas [82,83]; however, social support is necessary to help people integrate those healthier foods into their diet [84]. Adequate strategies are needed to address poorer dietary choices of individuals by educating about healthy cooking and snacking in general, and to further support nutritionally vulnerable populations in particular. 

### 4.4. Parents Interacting More with Their Children, but Also Being More Lenient

Parents interacting more with their children around food, including cooking, conversations, menu planning, gardening, and eating [32,33], was one of the most favorable outcomes of the pandemic crisis. Families enjoyed spending time together [29,32], and some parents also described that these moments became an opportunity for transmitting food-related knowledge [29]. Knowledge about food has been shown to influence food decisions [85,86] and inform meal planning [87], the latter being linked with an improved diet quality and less obesity [88]. Moreover, the importance of maternal nutrition knowledge on the diet quality of children/adolescents has been reported in several studies [89,90], including considerations for the mediating effect of the home environment [91]. Although people may use nutrition knowledge to change their eating behavior, this knowledge alone is unlikely to be effective [92], unless combined with the ability to apply it and motivation to change behavior [93]. For example, skills and knowledge on cooking may influence balanced food choices [94], whereas individuals with lower cooking skills are more likely to consume food away from home [95], which is often rich in energy, fat, and sugar, and lacks vegetables [71,72,95].

Parental food involvement is one of many different factors that shape the development of children’s food preferences and eating behaviors during the first years of life [96]; this is because caregivers act as powerful socialization agents in terms of both food providers and food models [97,98,99]. For example, evidence shows that parental food involvement predicts child preference or intake of fruits and vegetables [100,101], and may influence consumption of ‘healthy’ foods more than ‘unhealthy’ foods [100]; on the other hand, low food involvement has been associated with poor diet quality (low intakes of fruits and vegetables) in women [102], and lack of parental time has been attributed to one of the risk factors which can cumulatively lead to excess childhood weight gain [103]. Similarly, in one systematic review of parenting styles, feeding styles, feeding practices, and weight status in 4–12-year-old children, uninvolved, indulgent, or highly protective parenting has been associated with higher BMI, whereas authoritative parenting has been associated with a healthy BMI [104]. Moreover, involving children in food preparation had a positive effect on their eating behavior, as children would have more interest in and accepted certain foods more easily when they had helped to prepare them [29]. This included involving children in gardening [32,33], which may encourage taste testing and an increased fruit and vegetable intake in children [105]. Additionally, several experimental studies have shown that gardening is linked to lower obesity levels in adults [106,107], improves lifestyle sustainability [108,109], and may become a solution to address global warming [109]. Previously identified barriers to parent involvement include time poverty, lack of access, lack of financial resources, and lack of awareness [110]. Greater family interactions facilitated by the social lockdown should be preserved and further promoted by addressing barriers to parent involvement outside times of pandemic.

On the other hand, parents became more permissive when they changed their feeding practices during the COVID-19 pandemic. An indulgent feeding style, being characteristic of parents who encourage eating with few requests [111], has been associated with higher child BMI [104]. Permissive feeding style is one example of the specific parental feeding styles that may be affected by parent emotional distress [112], including parental stress during the lockdown [34]; for example, stress associated with the lockdown may be linked to child snack intake with potential impacts on child obesity risk [34]. Parents may also experience higher levels of stress and depressed mood as a result of food insecurity exacerbated by the COVID-19 crisis [113,114], which has been linked to parents who put pressure on their child to eat more to avoid wasting food that has been prepared [68]. Parental stress has been previously shown to result in poorer feeding practices [115], including differences between food secure and food insecure families [116]; for example, food insecurity has been associated with an increased use of restrictive feeding practices and pre-prepared foods, whereas parents who were food secure tended to respond with pressure-to-eat feeding practices and offer their children more fast-food [116]. Greater use of non-nutritive feeding during the lockdown was also related to soothing, especially with younger children [34]. Younger children require more guidance, including providing more structure around meals and restricting snacks [34], but instead are more often subjected to instrumental feeding and emotion-based snack feeding by their parents [34]. Mindful parenting can lower levels of parenting stress, leading to less frequent use of food as a reward, and therefore helping children break habits relating to disordered eating [115]. Stress management and educating parents about mindful child-feeding practices may encourage healthier eating behaviors among children/adolescents during future lockdowns, as well becoming a part of ongoing efforts to address dysfunctional parental practices around food.

### 4.5. New Trends in Food Shopping and Meal Planning

Some families experienced practical inconveniences with grocery shopping [29], including food shortages and increased prices [25,31]; others had concerns about social exposure, resulting in food stockpiling [37] or reduced travel frequency to shops [26,31]. In response to these new challenges in food shopping, other coping behaviors and behavioral adjustments included bulk buying [27] and “panic shopping” [31], as well as the adoption of meal planning skills [27,29] and online grocery shopping behaviors [26,27].

The pandemic poses major threats to global food security, including breaks in the food supply chain, food shortages and choice limitation, and food price spikes and volatility [117,118]. The resulting bulk purchasing and stockpiling were significantly correlated with increased food purchase, which in turn led to increased food waste [119]. Observed or perceived lack of resources due to COVID-19 also led to panic shopping, which, for some, has been viewed in positive terms as preparedness behaviors (e.g., to reduce future trips to shops) [120], but overall is a dangerous phenomenon given its effects on price increase, supply disruption, or store congestion [121,122]. Panic buying is mostly caused by consumers’ heightened anxiety and fear [122], which, during the pandemic, has been further reinforced by scarcity messages with limited quantity and time [123]. On the positive side, panic buying is a rare phenomenon [124], and when panic occurs, it only influences a small group of people for a short period of time [125]. More importantly, problems with food availability and increased prices were not universally experienced due to differences in market resilience [35]. For example, Chinese food availability scored higher than the U.S. because of the more versatile and diverse food retail sector in China, combined with proactive and progressive food security policies in urban planning implemented across the country [35]; on the other hand, food prices held steady in the U.S. as opposed to price volatility in China for reasons yet to be examined [35].

During the pandemic, many people also resorted to online shopping [126,127], which surged during the pandemic and eventually became unreliable [31], as retailers failed to keep pace with high continuous demand. For example, in India, the ‘stay at home’ regulation augmented the number of first-time users, who earlier were inhibited to shop online [128]. Common barriers to buying online include the security of transaction, the difficulty in using IT tools, and the quality of the delivery service, also linked to the characteristics of the product [129], whereas perceived the sustainability in purchasing online has been found to increase customer engagement [130]. During the pandemic, the shift to online shopping was caused by the closure of stationary retail stores [131] or concerns over COVID-19 (e.g., shopping inside grocery stores, avoiding public crowded gatherings) [132]. Despite many benefits of online shopping [133,134], experiences during the lockdown were mixed due to difficulty in finding delivery slots and incomplete food deliveries [31]. The long-term effects of the pandemic on online grocery shopping will require further analysis. It is possible that the digital-online shopping adoption becomes permanent [135], which, however, would have to be accompanied by grocers and retailers reidentifying their marketing strategies and enhancing their online shopping service to better serve online grocery shoppers; on the other hand, many online shoppers may choose to return to brick-and-mortar shopping when pandemic conditions subside, depending on customers’ intention and motivation for continuance usage of online shopping [132,136]. Nevertheless, online shopping seems to be the way forward in terms of promoting sustainability paths by decreasing the quantity of shopping trips [137], and thus achieving an ecological long-term stability in line with the 2030 Agenda’s sustainable development goals (SDGs) [138].

Finally, because of environmental effects of the COVID-19 pandemic on food security and food consumption, meal planning significantly improved. Benefits of meal planning for diet and health are multiple, including links with food consumption, diet quality, and weight status [88,139,140,141]. Specifically, planning meals in advance has been associated with increased frequencies of home meal preparation [139], having more family meals [142], a healthier diet and less obesity [88], and greater fruit and vegetable intakes [140], including the presence of fruits for dinner [141]. Moreover, meal planning has also helped successful weight losers to maintain their new weight [143], and could be a potential tool to offset time scarcity and reduce barriers of adherence to healthy eating [43]. During the pandemic, an increase in meal planning led to reduced household food waste [119,144], which was also correlated with behaviors focused on preserving foods, and using leftovers and shelf-stable items [119]. Private households have been identified as key actors in food waste generation [145], which has been attributed to resource depletion and greenhouse gas emissions [145]. It is therefore encouraging that efficient food use behaviors started during the pandemic may be continued, as shown by intentional declarations [119]. Moreover, it was also shown that even in times of pandemic characterized by food scarcity constraints, a palatable and diversified diet can be purchased very inexpensively from supermarkets, and visits to the supermarket can also be limited to one per month to reduce dangerous exposure, given effective meal planning is put in place [146]. These findings may inform future strategies relating to meal planning and waste management.

### 4.6. Uneven Burden of COVID-19 on Families

The impacts of COVID-19 on diet have not been felt uniformly across society. For poor families, the family’s food needs increased during the pandemic, and food-related support was critical, especially among single parents [31]. Changes in families’ home food environment and parent feeding practices, from before to during the pandemic, differed by food security status [36]. A greater increase of pressure to eat was found for parents with insecurity, who also reported more concerns about children being overweight due to increased food intake of high-calorie snack foods and desserts and sweets [36]. Deals and reduced-to-clear items remained an important part of families’ diet; however, infrequent shopping trips and reliance on unreliable supermarket home deliveries further compromised home food availability in fresh food products [31]. Moreover, single-parent families may have found it more challenging to adopt a healthy diet during the COVID-19 lockdown/s compared to parents living with partner [31]; for example, one study found that single parents had less time for meal provisioning at home because they had to entertain their children who otherwise would be at school [31]. One rapid review of qualitative evidence on parental perceptions of the food environment and their influence on food decisions among low-income families confirms that social support from families or government sources was an important first step in addressing health and nutritional inequities; however, long-term solutions are needed to tackle barriers to healthy eating, including child preferences, financial and time constraints, and location and access to food outlets [22]. 

People who are socioeconomically disadvantaged tend to have decreased access to healthy food retail outlets [147], such as supermarkets and grocery stores, and increased access to fast-food outlets where cheaper unhealthy food is readily available [148]. This has been known as a paradox of the obesity and poverty relationship that stems from both the easy availability and low cost of highly processed foods, in addition to unemployment and affordability constrains, lower education levels, and irregular meals in the population of poor people [149]. As affordability constraints remain an important determinant that relates to differences in obesity prevalence across geographical areas, it has been recommended that improving physical access to supermarkets and improving economic access to healthy foods are two valid strategies to deal with the obesity epidemic [150]. Recently, COVID-19 introduced new drivers of food insecurity, in addition to financial hardship faced by low-income households, by making access to food harder in terms of lack of food in the shops and through isolation [151]. As a result, in the UK, ‘a newly vulnerable group who were financially stable pre-COVID emerged, making reliance on overstretched food banks and food aid charities no longer a sustainable solution to food insecurity’ [151]. On the other hand, even in times of pandemic, a healthy diet can be maintained inexpensively through infrequent visits to the supermarket [146]. Nevertheless, for low-income families, making their eating habits more sustainable would require policy responses to low income, food access, and to the high cost of healthy foods [22].

### 4.7. Limitations

This review comprehensively examined the evidence relating to general parental food perspectives during the COVID-19 pandemic; however, it is not without its limitations. Studies retrieved using the search strategy were limited by the coverage of the search terms used, and their inclusion in the final synthesis was judgement by only one author. All eligible articles have been included and discussed; however, 14 papers that have been included in the final synthesis may not provide enough evidence to fully understand ongoing trends. Nevertheless, the results are an important first step in recognizing how this pandemic may be affecting the family food environment. It should also be noted that no papers were excluded from the final analysis on the basis of quality appraisal, which could have compromised the strength of this review’s findings; on the other hand, as all the relevant studies were included, this has likely contributed to a more well-rounded synthesis. Finally, as the research investigating the impact of the COVID-19 pandemic on the family food environment is still ongoing, there will be a need for a follow-up review capturing new trends.

## 5. Conclusions

The pandemic had profound impacts on household food dynamics, including both positive and negative changes, such as the increase in overall food intake comprised of regular home-cooked meals, on the one hand, and irregular snacking, on the other hand. Time became a factor that gave families the opportunity to enjoy interactions around food, but also led to boredom, straining family relationships and causing emotional overeating in children. It is therefore recommended that time should be better managed in the future to deal with time scarcity issues (e.g., through minimising time–income–space trade-offs faced by individuals, which would be a sustainable systematic solution) and to further promote and facilitate family engagement around food after the lockdown; moreover, educating parents and children on how to manage free time could help them prepare to cope with changes in future lockdowns. The pandemic also led to parental stress and a lenient parenting feeding style, which could be tackled by stress management and educating parents about mindful child-feeding practices, including healthy snacking. Household food security deteriorated among families with the lowest income, therefore requiring orchestrated policy responses to low income, food access, and to the high cost of healthy foods; however, effective meal planning may help overcome food scarcity constrains caused by the pandemic, leading to less food waste and healthier diet maintained inexpensively through infrequent visits to the supermarket. The switch towards online grocery shopping is encouraging given its ecological benefits, but the long-term effects of the pandemic on this recent trend in e-commerce will require further analysis. Similarly, the durability of other food-related changes caused by the pandemic, and how widespread they might be across large populations, deserves further considerations. 

## Figures and Tables

**Table 1:** Summary of results by research themes.

Study	Changes in Food Purchasing and Planning	Changes in Meal Preparation and Routines	Changes in Eating and Feeding Behaviors
Benson et al. [24]	Not addressed	Evidence of increases in parents’ time spent cooking and including children in cooking activities; higher intake of vegetables by parents who included children more frequently in cooking activities; the inclusion of children in cooking was associated with parental cooking skills confidence and provided life skills and increased diet quality	Not addressed
Carroll et al. [25]	Some concerns about grocery shopping, e.g., relating to stretching fresh produce to last longer	Spending more time cooking, making more meals from scratch, eating more meals with children, and involving children in meal preparation more often	Eating more food, including snack foods, and eating fewer foods from fast food and/or take out
Ferrante et al. [26]	Shopping less often, using online grocery shopping	Eating home-cooked meals more often	When eating out, parents were involved in deciding what their child eat, including nutrition concerns
Menon et al. [27]	Adoption of meal planning skills, increase in online food shopping, bulk buying, shortage of food items	Increased household cooking, involvement of children and male members in food-related activities, experimentation in the kitchen, reduced consumption of outside home food	Increase in overall food intake, including variety of home-cooked meals and snacking; health and immunity, family members’ preferences and taste, and food availability determined food choices
Philippe et al. [28]	Parents more frequently bought foods their child liked, but also more healthy and sustainable foods	Increased household cooking, more time cooking with their child(ren)	Child appetite and emotional overeating increased; parents became more permissive
Philippe et al. [29,30]	More fresh, seasonal, and local foods, paying more attention to the nutritional value of foods and meals, families have more time to plan meals and moments together	Cooking with the child was a pleasurable activity and an occasion to educate about food, to pass on certain cooking skills and values around food, and to taste new flavors	Spending more time together around food (home-made dishes, new recipes, cooking and eating together with the family at a calm pace); diversity of foods and balanced meals, but parents were also concerned about increased intake of palatable foods and weight gain
Sylvetsky et al. [30]	Stockpiling shelf stable foods due to grocery shortages and purchasing more sugary drinks (SDs) and snacks due to the whole family being at home	Making healthier choices because of not being “on the go” and cooking more meals at home, as opposed to eating out	Excess consumption of SDs and snacks among children; skipping breakfast when attending school virtually; parents removing prior restrictions on SDs and allowing more autonomy as a coping strategy to help children deal with change
Spyreli [31]	Food planning (ensuring healthy eating, less trips to the shop); food-related support was critical; some critical of bulk buying but in general stockpiling up on items; observing prices going up thus importance of food offers increased; shift towards home food deliveries and avoiding local shops; difficulty in finding delivery slots and incomplete food deliveries	Cooking more and healthier (home-made foods, increased intention to cook from raw ingredients); children getting involved in the kitchen	Trends in snacking behaviors; single-parent families may have found it more challenging to adopt a healthy diet
Nanayakkara [32]	Not addressed	Parents interacting more with their children about food, including cooking, menu planning, eating, conversations around food, and gardening; parents enjoyed preparing meals with their children	Eating hot and home-cooked food and more elaborate meals
Radwan et al. [33]	Parents sought their children’s opinions about what they would like to eat for the meals, who were also involved in menu planning	Parents interacting more with their children about food, including cooking, conversations, menu planning, gardening and eating	Eating more home-cooked or hot lunches; lunch quality improved, including increased variety, more elaborate and complex meals, and healthier meals; parents involved in controlling the food or snack intake of their children whose appetite increased
Jansen et al. [34]	Not addressed	More structure and positive interactions around food, including eating with or engaging with child around mealtimes; school-aged children were more likely to help prepare foods	Regular mealtimes and irregular snack times; more non-nutritive use of food and snacks because of stress (e.g., emotional and instrumental feeding); greater child intake frequency of sweet and savory snacks, with potential impact on child obesity and some evidence for mediation by snack parenting practices
Dou et al. [35]	More prudent use of food with less wastage; food prices held steady in U.S. but not in China; most foods were available, but many had limited options (U.S.); in China, all food types were well “stocked”, with some choice limitations	More time spend on food preparation and less eating out or ordering in	Overeating and increased eating frequency; overall, no change in weight, but people reporting weight gains outnumbered those reporting weight loss
Adams et al. [36]	Overall, the total amount of food in the home increased, including high-calorie snacks, desserts, and sweets, and non-perishable processed foods	Decrease in consumption of take-out/fast-food/already prepared meals and an increase in home-cooked meals	Restrictive feeding practices, pressure to eat, and monitoring; some parents cut or skipped meals; increase in non-perishable processed foods combined with concerns about child overweight; greater changes in parents’ concern about child overweight and pressure to eat observed for families experiencing food insecurity
Farello et al. [37]	Amount of food in the home increased because of “panic shopping”; desire for families to stock up on foods and minimize social exposure	Increase in home-cooked meals since parents spent more time at home	Increase in the consumption of high-calorie snack foods; the total amount of food in homes increased by 50%; more parents reported obesity in their children after lockdown

## Data Availability

Not applicable.

## Supplementary Materials

The following supporting information can be downloaded at: https://www.mdpi.com/article/10.3390/foods11182851/s1, Table S1: Additional characteristics of full-text studies included in the review.
